# Targeting EGFR and HER-2 with cetuximab- and trastuzumab-mediated immunotherapy in oesophageal squamous cell carcinoma

**DOI:** 10.1038/sj.bjc.6603885

**Published:** 2007-07-10

**Authors:** Y Kawaguchi, K Kono, K Mimura, F Mitsui, H Sugai, H Akaike, H Fujii

**Affiliations:** 1 First Department of Surgery, University of Yamanashi, 1110 Shimokato, Chuo-city, Yamanashi 409-3898, Japan

**Keywords:** cetuximab, trastuzumab, EGFR, HER-2, oesophageal cancer

## Abstract

We previously reported that oesophageal squamous cell carcinoma (SCC) had a relatively high incidence of EGFR and HER-2 overexpression. Thus, anti-HER family targeting may become a promising approach to treat oesophageal SCC. In the present study, we investigated (a) the distribution of EGFR and HER-2 expression in oesophageal SCC (*n*=66) detected by immunohistochemistry and (b) cetuximab- and/or trastuzumab-mediated biological activity (antiproliferative effect by the MTT assay, apoptosis-inducing activity by the annexin V/propidium iodide assay, and antibody-dependent cellular cytotoxicity (ADCC) by the ^51^Cr-release assay) against oesophageal SCC cell lines with various levels of EGFR and HER-2. Twelve of the 66 patients (18%) showed both EGFR- and HER-2 expression. Out of both EGFR- and HER-2-positive cases, nine cases (75%) showed EGFR and HER-2 expression in individually distinct regions. Furthermore, the combination of cetuximab and trastuzumab could induce synergistic antiproliferative effects and additional ADCC activities against not all, but several oesophageal SCC cell lines with EGFR and HER-2 expression. The combination of cetuximab and trastuzumab may be useful in the treatment of oesophageal SCC with EGFR and HER-2 expression.

Oesophageal cancer is the sixth most frequent cause of cancer death worldwide (Pisani *et al*, 1993). Most patients with oesophageal cancer in Japan have squamous cell carcinoma (SCC), while most of those in Western countries have adenocarcinoma. Despite improvements in surgical techniques and perioperative management (Akiyama *et al*, 1994; Ando *et al*, 2000), and surgery combined with chemotherapy (Ando *et al*, 2003) and/or radiotherapy (Ishikura *et al*, 2003), the prognosis remains poor. Therefore, for oesophageal SCC patients, novel therapies such as molecular-targeted therapy, including small molecule inhibitors of tyrosine kinases or humanised monoclonal antibodies, are very much needed.

The HER family of receptor tyrosine kinases consists of four members: EGFR (HER-1), HER-2, HER-3, and HER-4. The HER family-related signalling is reported to play an important role in modulating cell proliferation, survival, migration, and differentiation. Despite the large number of ligands so far identified for EGFR and HER-3 and -4, no direct ligand for HER-2 has yet been discovered. As HER-2 is the preferred heterodimerisation partner for all other HER family members, increasing evidence suggests that the primary function of HER-2 is as a co-receptor (Tzahar *et al*, 1996; Graus-Porta *et al*, 1997). For example, when ligands such as EGF bind to EGFR, EGFR is heterodimerised with HER-2, leading to the subsequent activation of EGFR tyrosine kinase.

Overexpression of the HER family members has been identified in a variety of human cancers such as gastrointestinal tract (Ghaderi *et al*, 2002; Kimura *et al*, 2004), colorectal (Hayashi *et al*, 1994; Porebska *et al*, 2000; Ooi *et al*, 2004), breast (Suo *et al*, 2002; Witton *et al*, 2003; Abd El-Rehim *et al*, 2004; DiGiovanna *et al*, 2005), lung (Hirsch *et al*, 2003), prostate (Di Lorenzo *et al*, 2002), and bladder cancers (Chow *et al*, 2001; Wester *et al*, 2002), and is correlated in a wide variety of tumours with the progression (Yarden and Sliwkowski, 2001). In particular, we and others have recently reported that the overexpression of EGFR of oesophageal SCC, partially accounted for by gene amplification, is found in 50–70% (Itakura *et al*, 1994; Hanawa *et al*, 2006), and is indicative of a poor prognosis (Ozawa *et al*, 1989; Yano *et al*, 1991). Moreover, we showed that the overexpression of HER-2 in oesophageal SCC was found in 30.3% (Mimura *et al*, 2005b). These results indicated that oesophageal SCC shows a relatively high incidence of EGFR and/or HER-2 overexpression.

There are several potential strategies for anti-HER family targeting. Two anti-HER family-targeting therapies that have been in clinical development are small-molecule EGFR tyrosine kinase inhibitors such as gefitinib (Ranson *et al*, 2002; Fukuoka *et al*, 2003) and humanised antibodies against the HER family represented by cetuximab and trastuzumab (Herbst and Hong, 2002; Needle, 2002). There are many mechanisms that are thought to contribute to the antitumour activity of cetuximab and trastuzumab, including a direct inhibition of EGFR tyrosine kinase activity (Sato *et al*, 1983; Sliwkowski *et al*, 1999), the inhibition of cell cycle progression (Wu *et al*, 1995; Peng *et al*, 1996), and increased levels and activities of pro-apoptotic molecules (Wu *et al*, 1995; Cuello *et al*, 2001; Liu *et al*, 2001). We recently reported the application of trastuzumab for oesophageal SCC with the analysis of antibody-dependent cellular cytotoxicity (ADCC) mediated by trastuzumab (Mimura *et al*, 2005a). These results encourage us to apply a combination therapy of cetuximab and trastuzumab for oesophageal SCC, aiming at a synergistic effect. Thus, it is important to identify how expressions of EGFR and HER-2 are distributed in oesophageal SCC and if the combination of cetuximab and trastuzumab has a synergistic antitumour effect against oesophageal SCC.

In the present study, we investigated (a) the distribution of EGFR and HER-2 expression in oesophageal SCC detected by immunohistochemistry (IHC) and (b) the biological activity (antiproliferative effect, apoptosis-inducing activity, and ADCC) of cetuximab and trastuzumab against oesophageal SCC cell lines with various levels of EGFR and HER-2.

## MATERIALS AND METHODS

### Patients

We examined 66 cases of primary oesophageal SCCs that were histologically diagnosed and treated in the First Department of Surgery, University of Yamanashi Hospital. The patients had not received irradiation or chemotherapy before surgery. All patients had undergone oesophagectomy with two-field (*n*=39) or three-field (*n*=27) lymph node dissection between 1994 and 1999. The patients were classified using the tumour node metastasis (TNM) classification. The characteristics of the patients are shown in Table 1. The study was approved by the Ethical Committee of the University of Yamanashi, and written informed consent was obtained from all individuals.

### Cell lines and fresh tumour samples

Oesophageal SCC cell lines TE3, TE4, and TE5 were a kind gift from Dr Nishihara (Institute of Development, Aging and Cancer, University of Tohoku, Sendai, Japan). The oesophageal SCC cell line KYSE50 was purchased from the Health Science Research Resources Bank (Osaka, Japan). All cells were cultured in RPMI 1640 medium with 5% fetal bovine serum, 100 units ml^−1^ penicillin, 100 *μ*g ml^−1^ streptomycin, and 2 mmol l^−1^ L-glutamine.

Primary solid tumour from oesophageal SCC patients (*n*=6) was isolated during surgery and was homogenised by mechanical mincing. Then, cell mixtures were passed through a cell strainer (Becton Dickinson Labware, Franklin Lakes, NJ, USA) and suspended as a single-cell suspension. A single-cell suspension derived from solid tumours and malignant pleural effusion was purified by centrifugation with Ficoll–Paque (Pharmacia, Uppsala, Sweden).

### Chemicals and antibodies

Humanised mouse anti-human EGFR antibody, cetuximab (Erbitux™), was purchased from Merck (Dietikon, Switzerland). The anti-HER-2 monoclonal antibody trastuzumab (Herceptin™) and anti-CD20 mAb rituxan, which is an isotype-matched control mAb, were purchased from Roche (Basel, Switzerland).

### Immunohistochemistry

All resected oesophageal samples were immediately immersed in 20% buffered neutral formalin, fixed overnight, and embedded in paraffin according to standard procedures.

To detect EGFR, a paraffin-embedded tissue specimen was sectioned at 4 *μ*m thickness and immunohistochemically stained by the labelled streptavidin biotin (LSAB) method. After deparaffinisation and rehydration, the sections were autoclaved in 0.01 M citrate buffer (pH 7.0) at 121°C for 10 min. Then, the sections were cooled at room temperature for 60 min, immersed in 3% hydrogen peroxidase for 10 min to block endogenous peroxidase activity, and then washed in phosphate-buffered saline (PBS) for 5 min. To detect EGFR, mouse anti-human EGFR mAb (DakoCytomation, Glostrup, Denmark) was used. The sections were incubated with the antibody (diluted 1 : 40) for 24 h at 4°C in a moist chamber. After washing three times with PBS for 5 min, the sections were reacted with the secondary antibody (biotinylated anti-mouse antibody) for 30 min at room temperature. Then the sections were washed again three times with PBS for 5 min after which they were reacted with peroxidase-conjugated streptavidin for 30 min at room temperature. After this, the sections were washed again three times with PBS for 5 min and were reacted with a solution containing 0.06 mM 3,3′-diaminobenzidine and 2 mM hydrogen peroxide in 0.05% Tris–HCl buffered at pH 7.6 for 10 min. They were then counterstained with haematoxylin for 30 s. After dehydrating with 60–100% isopropyl alcohol, penetrating, and mounting, the sections were observed.

To detect HER-2, immunohistochemical staining was performed using the HercepTest (DakoCytomation) according to the recommendations of the manufacturer. Deparaffinised and rehydrated tissue sections were incubated with the Epitope Retrieval Solution in a hot water bath for 40 min at 95–99°C. Then, the sections were cooled to room temperature for 20 min, washed with Tris buffer for 5 min, and endogenous peroxidase was blocked with 3% hydrogen peroxide for 5 min. The primary antibody was rabbit polyclonal antibody to human HER-2, which recognises an intracytoplasmic part of HER-2, and the primary negative control antibody was an immunoglobulin fraction of normal rabbit serum at an equivalent protein concentration to the antibody to HER-2. The sections were washed with TRIS buffer for 5 min and incubated with the primary antibody or the primary negative control antibody at room temperature for 30 min. After rewashing with Tris buffer for 5 min twice, the primary antibody was detected by incubating at room temperature for 30 min using the visualisation reagents, dextran polymer conjugated with horseradish peroxidase and affinity-isolated goat anti-rabbit immunoglobulin. Subsequently, following the rewashing with Tris buffer for 5 min twice, diaminobenzidine was added as a visualisation reagent for 10 min and sections were counterstained with haematoxylin.

EGFR or HER-2 positivity in the IHC analysis was carried out by three observers (YK, FM, and KK). The intensity of reactivity was scored using four categories: negative, no discernible staining or background type staining; 1+, definite cytoplasmic staining and/or equivocal discontinuous membrane staining; 2+, unequivocal membrane staining with moderate intensity; and 3+, strong and complete plasma membrane staining.

### Flow cytometry

For the analysis of EGFR expression by flow cytometry, mouse anti-human EGFR mAb (DakoCytomation) as the primary mAb and an FITC-conjugated polyclonal rabbit anti-mouse mAb as the secondary mAb (DakoCytomation) were used, and for the analysis of HER-2 expression, a phycoerythrin-labelled anti-HER-2/neu mAb (Becton Dickinson, San Jose, CA, USA) was used. As a negative control for the primary mAb, mouse immunoglobulin G1 mAb (Beckman-Coulter, Miami, FL, USA) was used.

Each step of the incubation with mAb was performed at 4°C for 30 min. After cells were washed twice in PBS, the stained cells were analysed on a flow cytometer.

### Fluorescence *in situ* hybridisation analysis

Fluorescence *in situ* hybridisation (FISH) analysis was carried out using the PathVysion HER-2 DNA Probe Kit (Vysis, Downers Grove, IL, USA). The HER-2/neu-SpectrumOrange probe contains a DNA sequence specific for the HER-2 gene locus (17q11.2–q12). The chromosome enumeration probe 17 (CEP 17)/SpectrumGreen probe contains alpha-satellite DNA that hybridises to the D17Z1 locus (centromere region of chromosome 17). To determine the copy number for chromosome 17, we used CEP 17 as the control. Fluorescence *in situ* hybridisation procedures were conducted according to the guidelines of the manufacturer, except the removal of the protein from the tissue sections as described previously (Takehana *et al*, 2002). In brief, deparaffinised and dehydrated sections were incubated in 20% sodium bisulphate/2 × standard saline citrate (SSC) at 43°C for 20 min. After being washed with SSC, sections were treated with proteinase K (Boehringer-Mannheim, Mannheim, Germany) at 37°C for 25 min. Subsequently, denaturation, hybridisation, and post-hybridisation washing were carried out according to the manufacturer’s protocol, and the sections were counterstained with 4′,6-diamidine-2′-phenylindole dihydrochloride (Oncor, Gaithersburg, MD, USA). Fluorescence *in situ* hybridisation analysis was performed using a fluorescence microscope (Olympus, Tokyo, Japan) equipped with Triple Bandpass Filter sets (Vysis). Signals were countered for at least 40 cancer nuclei per tumour. In accordance with our previous studies with FISH, a cell was considered to show amplification when a definite cluster or more than 10 orange signals of HER-2 were observed (Takehana *et al*, 2002).

### Antibody-dependent cell-mediated cytotoxicity assay

Peripheral blood mononuclear cells (PBMC) were separated from peripheral blood obtained from healthy donors and oesophageal SCC patients before treatment by centrifugation with Ficoll–Paque (Pharmacia). After the target cells were labelled with 50 *μ*Ci of ^51^Cr for 60 min, target cells (5 × 10^3^ well^−1^) and PBMC from healthy donors or oesophageal cancer patients as effector cells were co-incubated at various effector/target ratios in 200 *μ*l of X-VIVO medium in a 96-well U-bottomed plate in triplicate with indicated doses of cetuximab or/and trastuzumab or a control antibody, rituxan. After 6 h of incubation, the radioactivity of the supernatant (100 *μ*l) was measured with a *γ*-counter. The percentage of specific lysis=100 × (experimental c.p.m.−spontaneous c.p.m.)/(maximum c.p.m.−spontaneous c.p.m.).

### Apoptosis

Each cell line (2 × 10^5^ cells) was incubated in 2 ml of X-VIVO with control mAb alone, cetuximab (0.5 *μ*g ml^−1^) alone, trastuzumab (10 *μ*g ml^−1^) alone, and cetuximab in combination with trastuzumab at 37°C in a six-well plate. After incubation for 24 h, apoptosis in each cell line was measured by staining with FITC-conjugated annexin-V and propidium iodide (PI) using a MEBCYTO Apoptosis kit (MBL, Nagoya, Japan) following the manufacturer's recommendations.

### MTT cell proliferation assay

Each cell line (2500 cells) was incubated in 200 *μ*l of X-VIVO with indicated doses of cetuximab or/and trastuzumab, or a control antibody in a 96-well flat-bottomed plate in triplicate. After incubation for 96 h at 37°C, 50 *μ*l of MTT (3-(4,5-dimethylthiazol-2-yl)-2,5-diphenyltetrazolium bromide, 2 mg ml^−1^; Sigma, St Louis, MO, USA) was added to each well and incubation was carried out for 4 h. Then, the supernatant was discarded and the crystal products were eluted with DMSO (50 *μ*l well^−1^; Sigma). Colorimetric evaluation was performed using a spectrophotometer at 570 nm. The inhibition of proliferation was shown as % cell growth inhibition induced by cetuximab or/and trastuzumab in comparison with that induced by control mAb.

### Survival analysis

Actuarial overall survival rates were analysed by the Kaplan–Meier method, and survival was measured in months from operation to death or the last review. The log-rank test was applied to compare the two groups.

### Statistics

To evaluate significant differences between groups, Student's *t*-test was performed. Significance was considered at *P*<0.05.

## RESULTS

### Frequencies and patterns of EGFR and HER-2 expression in oesophageal SCC

**Figure 1:**
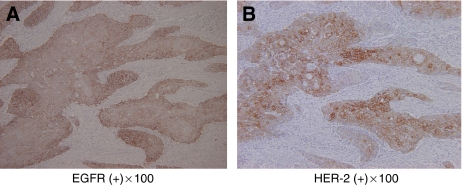
Immunohistochemical staining of EGFR and HER-2 in oesophageal SCC. In oesophageal SCC, EGFR and HER-2 expressions were evaluated by IHC in the serial sections. Representative stainings for EGFR (**A**) and HER-2 (**B**) are shown, and both expressions were seen in the same regions of the oesophageal SCC tumour ( × 100).

**Figure 2:**
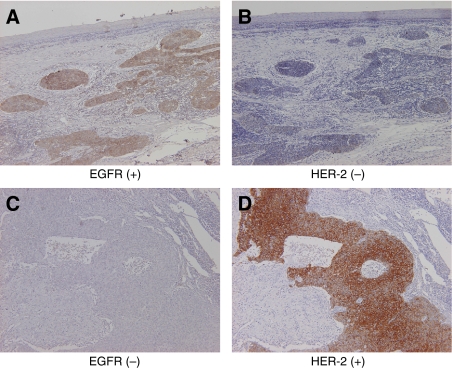
Immunohistochemical staining of EGFR and HER-2 in oesophageal SCC. In oesophageal SCC, EGFR and HER-2 expressions were evaluated by IHC in the serial sections ( × 100). The region with EGFR-positive expression (**A**) was negative for HER-2 (**B**) in the same tumour. On the contrary, the region with HER-2-positive expression (**D**) was negative for EGFR (**C**) in the same tumour.

Sixty-six oesophageal SCC tumours were examined for both EGFR and HER-2 expression in serial sections by IHC. EGFR-positive expression was observed in 22 cases (33%), while HER-2-positive expression was noted in 20 cases (31%). Both EGFR and HER-2 expressions in the same patients were observed in 12 cases (18%) (Figures 1 and 2), in which expressions of both EGFR and HER-2 were seen in the same tumour regions in two cases (Figure 1), and EGFR and HER-2 expressions were seen in individually distinct regions in nine cases (Figure 2). The data of EGFR and HER-2 expression in oesophageal SCC indicated that 45% of the patients showed either EGFR or HER-2 expression, and 18% of the patients showed both EGFR and HER-2. Of both EGFR- and HER-2-positive cases, 75% showed EGFR and HER-2 expression in individually distinct regions.

Furthermore, distribution of the grading of IHC for EGFR and HER-2 is shown in Table 2, indicating that there were variable patterns in their grading.

### Oesophageal SCC patient survival in relation to EGFR and HER-2 expression

**Figure 3:**
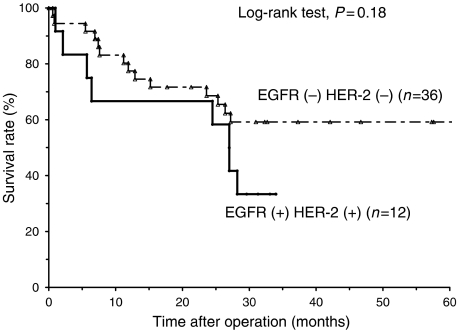
Survival curves of the patients with oesophageal SCC in relation to EGFR and HER-2 expression. Actuarial overall survival rates were analysed by the Kaplan–Meier method, and survival was measured in months from operation to death or the last review.

There was a tendency that the survival rate of patients with both EGFR (+) and HER-2 (+) was lower than those with EGFR (−) and HER-2 (−), although it was not significant (Figure 3).

### Synergistic antiproliferative effect of cetuximab and trastuzumab against oesophageal SCC

We have reported previously six different oesophageal SCC cell lines with variable expressions of HER-2 (Mimura *et al*, 2005a). Of these, TE3 was selected as a low-HER-2- and high-EGFR-expressing cell, KYSE50 and TE5 as a moderate-HER-2- and moderate-EGFR-expressing cell, and TE4 as a high-HER-2- and low-EGFR-expressing cell in the present study analysed by flow cytometry (Table 3). In FISH analysis, HER-2 gene amplification was seen in TE4 (Table 3), and polysomy, in which cancer nuclei showed more than three HER-2 signals accompanied with the same number of centromere 17 signals, was seen in TE3, TE5, and KYSE50 (Table 3). There was no significant quantitative correlation between HER-2 and EGFR expression analysed by flow cytometry in six different oesophageal SCC cell lines (data not shown).

**Figure 4:**
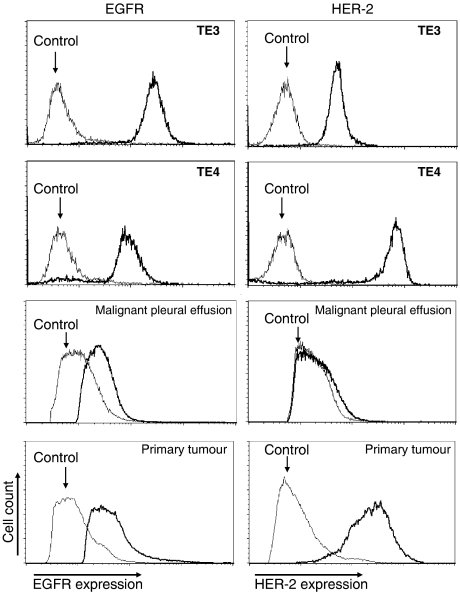
The degree of EGFR and HER-2 expression on oesophageal SCC cell lines and freshly isolated SCC tumours. EGFR and HER-2 expression was evaluated by flow cytometric analysis on oesophageal SCC cell lines and freshly isolated SCC tumours derived from two different patients.

Next, we examined HER-2 and EGFR expression in freshly isolated tumours (primary tumour and malignant pleural effusion) derived from six different oesophageal SCC patients. Representative flow data revealed the weak or moderate, but significant levels of HER-2 and EGFR expression in comparison with oesophageal SCC cell lines analysed by flow cytometry (Figure 4).

**Figure 5:**
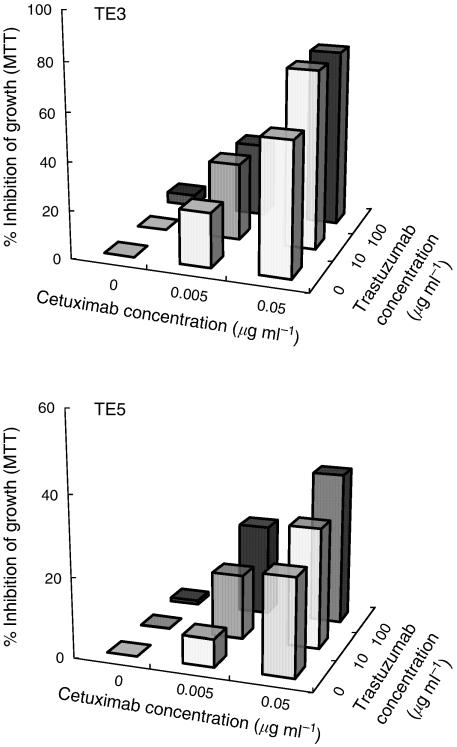
Synergistic antiproliferative effects of cetuximab and trastuzumab for oesophageal SCC cell lines. The oesophageal SCC lines, TE3 (HER-2 low and EGFR high) and TE5 (HER-2 moderate and EGFR moderate), were analysed by the MTT assay in various dose combinations of cetuximab and trastuzumab. The inhibition of proliferation was shown as % cell growth inhibition induced by cetuximab and/or trastuzumab in comparison with that induced by control mAb.

To examine the antiproliferative activity of cetuximab and/or trastuzumab, the MTT assay was performed. Of note, synergistic antiproliferative effects of cetuximab and trastuzumab were observed in TE3 (HER-2 low and EGFR high), while an additional antiproliferative effect was seen in KYSE50 (Table 3). Furthermore, the synergistic antiproliferative effects were confirmed in variable dose combinations of cetuximab and trastuzumab, and the same effects were also observed in the different oesophageal SCC TE5 (HER-2 moderate and EGFR –moderate; Figure 5). To examine the apoptosis-inducing activity of cetuximab and/or trastuzumab, the annexin–PI assay was performed. There were marginal levels of apoptosis induced by cetuximab and/or trastuzumab (Table 3).

### Cetuximab- and/or trastuzumab-mediated ADCC for oesophageal SCC

**Figure 6:**
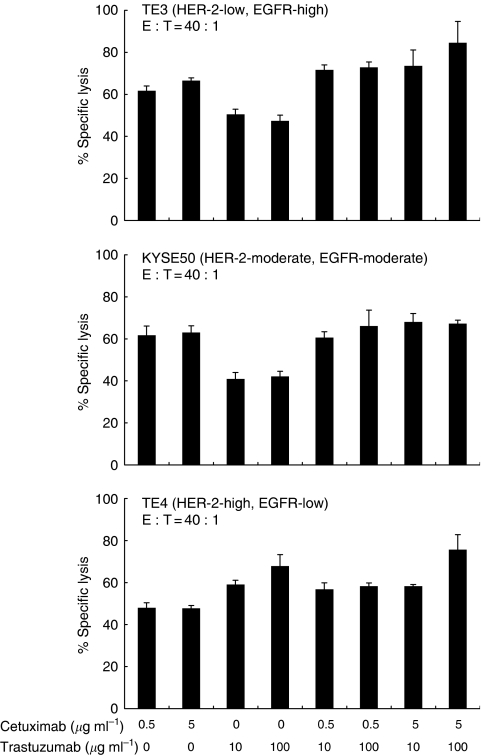
Cetuximab- and/or trastuzumab-mediated ADCC for oesophageal SCC cell lines. Oesophageal SCC cell lines with various levels of EGFR and HER-2 expression (TE3, KYSE50, and TE4) were analysed for ADCC derived from healthy donor’s PBMC in various dose combinations of cetuximab, trastuzumab, and control mAb by the 6-h ^51^Cr release assay. E : T, effector/target ratios.

**Figure 7:**
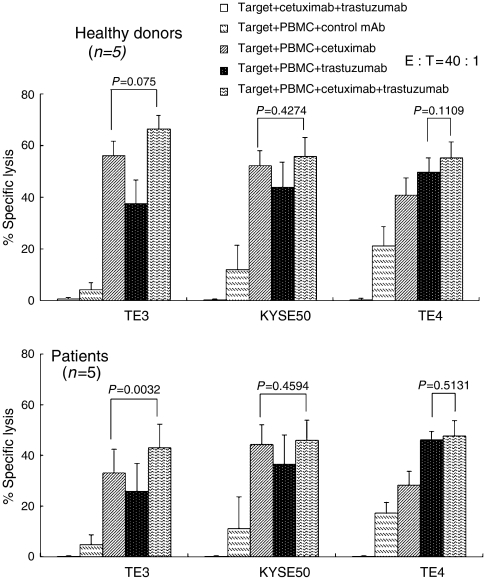
Cetuximab- and/or trastuzumab-mediated ADCC for oesophageal SCC in healthy donors and oesophageal SCC patients. Oesophageal SCC cell lines with various levels of EGFR and HER-2 expression were analysed for ADCC by healthy donors’ PBMC and patients’ PBMC in the presence of cetuximab (0.5 *μ*g ml^−1^) and/or trastuzumab (10 *μ*g ml^−1^) or control mAb (1 *μ*g ml^−1^) by the 6-h ^51^Cr release assay. Summarised data from healthy donors’ PBMC (*n*=5) and oesophageal cancer patients’ PBMC (*n*=5) are shown. E : T, effector/target ratios.

Next, we investigated whether the combination of cetuximab and trastuzumab induces synergistic effects in ADCC against oesophageal SCC with different levels of EGFR and HER-2. Representative data with several dose combinations of cetuximab and trastuzumab induced very marginal enhancements of ADCC derived from healthy donor’s PBMC against oesophageal SCC (Figure 6). Summarised data using PBMC from healthy donors (*n*=5) and oesophageal cancer patients (*n*=5) showed that marginal additional effects of cetuximab and trastuzumab were found only in ADCC for TE3, while no significant effects for KYSE50 or TE4 were seen (Figure 7).

## DISCUSSION

The present study contains important findings relevant to EGFR- and HER-2-targeted therapy for oesophageal SCC. First, 30 out of 66 patients (45%) showed either EGFR or HER-2 expression, and 18% of the patients showed both EGFR and HER-2. Second, the combination of cetuximab and trastuzumab could induce synergistic antiproliferative effects against not all, but several oesophageal SCC cell lines with EGFR and HER-2 expression.

Activation of the HER family triggers a network of signalling pathways related to tumour cell proliferation and migration (Yarden and Sliwkowski, 2001). A number of strategies against EGFR and HER-2 have been developed, including mAbs and small-molecule kinase inhibitors (Mendelsohn and Baselga, 2003). There have been data from clinical trials demonstrating the results of applying anti-EGFR tyrosine kinase inhibitors (gefitinib or erlotinib) to oesophageal adenocarcinoma (Dragovich *et al*, 2006; Kwak *et al*, 2006), indicating that erlotinib may be active in patients with oesophageal adenocarcinoma and the useful molecular marker will be needed to predict the therapeutic response.

Although there is no previous report describing the clinical application of cetuximab to oesophageal SCC patients, it has been shown that cetuximab-induced antitumour activity did not correlate directly with the levels of EGFR expression in human tumour xenograft models (Wild *et al*, 2006). These observations suggest that molecular events other than EGFR levels are true determinants of *in vivo* responsiveness to EGFR-targeted therapy, and further investigation is necessary to find out the factor that affects the antitumour effect of cetuximab and gefitinib.

We recently reported that treatment with trastuzumab could induce antitumour activities against oesophageal SCC with HER-2 expression, mainly mediated by ADCC activity. However, the trastuzumab-mediated ADCC activities reflected the degree of HER-2 expression, and furthermore, patients’ PBMC-derived ADCC was impaired in comparison to healthy donors. These results suggested that some modalities aiming at enhancing the trastuzumab-mediated antitumour effect are needed for the successful treatment of oesophageal SCC with trastuzumab. One possible strategy to enhance antitumour activities is a combination of trastuzumab with anti-EGFR mAb, cetuximab.

With regard to the expression of HER-2 and EGFR on oesophageal SCC, EGFR-positive tumours were observed in 35% of patients with oesophageal SCC, and HER-2-positive tumours were observed in 31% of them in the present study, in line with previous reports (Mimura *et al*, 2005b). This result was the high-end of the data in literature showing HER-2 expression in oesophageal SCC varying between 0 and 31% (Sunpaweravong *et al*, 2005), in which the different rate of HER-2 expression seems to be due to the different criteria for evaluating the results. Importantly, both EGFR- and HER-2-positive tumours were observed in 18% of all patients, out of which 75% showed EGFR and HER-2 expression in individually distinct regions. These *in vivo* data suggest that combined targeting with EGFR and HER-2 may result in an additional clinical response in patients with both EGFR and HER-2 expression.

Both antibodies were reported to have functions including internalisation and downregulation of the receptors (Fan *et al*, 1994; Goldstein *et al*, 1995; Sliwkowski *et al*, 1999), inhibition of tyrosine kinase activity (Sato *et al*, 1983), inhibition of cell cycle progression (Wu *et al*, 1995; Peng *et al*, 1996), and increased levels and activities of pro-apoptotic molecules (Wu *et al*, 1995; Liu *et al*, 2001). In addition, we recently reported that trastuzumab could induce ADCC activity for oesophageal SCC (Mimura *et al*, 2005a) or gastric cancer (Kono *et al*, 2002).

It has been shown that treatment with cetuximab or trastuzumab for breast cancer cells promoted the specific induction of pro-apoptotic molecules and resulted in the upregulation of chemosensitisation (Real *et al*, 2005). Furthermore, it has been reported that EGFR-HER-2 heterodimers are rate-limiting in the EGF-mediated proliferation of tumour cells (Hsieh *et al*, 2000). These results suggested that EGFR and HER-2 may interact with each other and lead to effective antitumour activity. As a novel and important finding in the present study, the combination of cetuximab and trastuzumab could induce synergistic antiproliferative effects against several oesophageal SCC cell lines with EGFR and HER-2 expression. However, the levels of EGFR and HER-2 expression in oesophageal SCC cell lines was not the only factor predicting the sensitivity to cetuximab and trastuzumab, since SCC cell lines, such as KYSE50 and TE5, with almost the same level of EGFR and HER-2 –expression, had a different amount of synergistic, antiproliferative effects with cetuximab and trastuzumab. Further investigation is necessary to elucidate the factors that affect the antitumour effect of cetuximab and trastuzumab combination.

In conclusion, the combination of cetuximab and trastuzumab could induce synergistic antiproliferative effects and additional ADCC activities against several oesophageal SCC cells. A better understanding of the detailed mechanisms involved in EGFR and/or HER-2 may help identify new therapeutic targets in oesophageal SCC.

## Figures and Tables

**Table 1:** Clinical features of the patients (*n*=66)

*Age (years)*	
Mean	65.3
Range	45–81
	
*Gender*	
Male	62
Female	4
	
*Primary tumour* a	
pTis	2
pT1a	8
pT1b	19
pT2	5
pT3	32
	
*Lymph node metastasis*	
Negative	29
Positive	37
	
*Histological grade*	
Well differentiated	16
Moderately differentiated	35
Poorly differentiated	15
	
*Stage* a	
0	9
I	5
II	26
III	19
IVa	6
IVb	1

a The grade of tumour and stages were defined according to the UICC (TNM) classification.

**Table 2:** Grading patterns of HER-2 and EGFR expression in oesophageal SCC (*n*=66)

		**IHC scores of HER-2**				
		**3+**	**2+**	**1+**	**0**	**Total**
IHC scores of EGFR	3+	2	3	1	5	11
	2+	0	0	4	4	8
	1+	1	0	1	1	3
	0	0	3	5	36	44
						
	Total	3	6	11	46	66

IHC=immunohistochemistry; SCC=squamous cell carcinoma.

**Table 3:** Antitumour effect against oesophageal cancer cell lines

	**TE3**	**TE5**	**KYSE50**	**TE4**
*EGFR and HER-2 status*				
EGFR FACS (MFI)	211	150	114	76
HER-2 FACS (MFI)	46	113	83	258
HER-2 FISH	Polysomy	Polysomy	Polysomy	Cluster
				
% *Inhibition of growth (MTT)*				
Ce (0.5 *μ*g ml^−1^)	55.1±1.1	21.7±1.3	33.5±3.8	−3.8±5.2
Tra (10 *μ*g ml^−1^)	−2.2±5.4	2.8±5.4	16.8±0.7	9.7±3.6
Ce+Tra	74.3±2.4	32.9±2.2	39.8±4.1	4.1±3.1
				
*Apoptosis* (%)				
Medium	4.7±1.4	3.2±0.6	7.8±0.3	8.2±0.7
Medium+Ce (0.5 *μ*g ml^−1^)	8.7±0.6	5.0±0.5	9.1±0.7	9.8±1.6
Medium+Tra (10 *μ*g ml^−1^)	4.2±0.6	2.5±0.1	8.5±2.3	10.0±0.7
Medium+Ce+Tra	13.7±2.3	5.3±0.3	11.1±0.5	11.5±2.2

Cet=cetuximab; FACS=fluorescence-activated cell sorting; FISH=Fluorescence *in situ* hybridisation; MFI=mean fluorescence intensity; MTT=3-(4,5-dimethylthiazol-2-yl)-2,5-diphenyltetrazolium bromide; Tra=trastuzumab.
